# Exploring the Regulatory Role of Circular RNAs in Neurodegenerative Disorders

**DOI:** 10.3390/ijms20215477

**Published:** 2019-11-04

**Authors:** Eleonora D’Ambra, Davide Capauto, Mariangela Morlando

**Affiliations:** 1 Center for Life Nano Science@Sapienza, Istituto Italiano di Tecnologia, 00161 Rome, Italy; eleonora.dambra@uniroma1.it; 2 Department of Biology and Biotechnology Charles Darwin, Sapienza University of Rome, 00185 Rome, Italy; davidecapauto@gmail.com; 3 Department of Pharmaceutical Sciences, “Department of Excellence 2018-2022”, University of Perugia, 06122 Perugia, Italy

**Keywords:** circRNAs, nervous system, neurodegenerative disease, ALS, Alzheimer, Parkinson, Biomarkers

## Abstract

Circular RNAs (circRNAs) are a distinctive class of regulatory non-coding RNAs characterised by the presence of covalently closed ends. They are evolutionary conserved molecules, and although detected in different tissues, circRNAs resulted specifically enriched in the nervous system. Recent studies have shown that circRNAs are dynamically modulated during neuronal development and aging, that circRNAs are enriched at synaptic levels and resulted modulated after synaptic plasticity induction. This has suggested that circRNAs might play an important role in neuronal specification and activity. Despite the exact function of circRNAs is still poorly understood, emerging evidence indicates that circRNAs have important regulatory functions that might extensively contribute to the dynamic modulation of gene expression that supports neuronal pathways. More interestingly, deregulation of circRNAs expression has been linked with various pathological conditions. In this review, we describe current advances in the field of circRNA biogenesis and function in the nervous system both in physiological and in pathological conditions, and we specifically lay out their association with neurodegenerative diseases. Furthermore, we discuss the opportunity to exploit circRNAs for innovative therapeutic approaches and, due to their high stability, to use circRNAs as suitable biomarkers for diagnosis and disease progression.

## 1. Introduction

In the last decade, fast advances in the resolution of high-throughput technologies dramatically improved our understanding of transcriptome diversity of complex organisms [1]. Nowadays, we are indeed fully aware that a considerable portion of the mammalian genome gives rise to a diverse family of transcripts with no coding capacity, the non-coding RNAs (ncRNAs), exerting structural and/or regulatory functions (Table 1). Circular RNAs (circRNAs) are recently identified molecules belonging to this latter class of ncRNA transcripts. They are particularly stable, due to their covalently closed structure, they are evolutionary conserved and expressed in a tissue-specific manner, but resulted particularly enriched in the nervous system [2,3,4]. In particular, circRNAs expression is regulated during neuronal differentiation and nervous system development and modulated by synaptic activity [3,4] suggesting that, as regulatory molecules, they might contribute to the fine-tuned and well-orchestrated control of gene expression during neuron specification and function. Indeed, even though not much is known about circRNA mode of action, the few species characterized have been shown to act as molecular decoy for microRNAs (miRNAs) or RNA binding proteins (RBPs) [3,5,6,7,8,9,10,11], to control transcription of their host genes [12,13,14] and, although classify as ncRNAs, some of them hold the capacity to direct synthesis of short peptides/proteins [15,16,17,18,19,20]. This strongly supports the hypothesis that neuronal cells might also rely on the activity of these transcripts to precisely regulate gene expression in time and space.

Currently, dysregulation of RNA metabolism appears to be an important contributor to the pathogenesis of diverse neurodegenerative disorders, such as Amyotrophic lateral sclerosis (ALS), Alzheimer’s (AD) and Parkinson’s disease (PD) [21]. Indeed, mutations of several genes encoding for RBPs are linked to the pathogenesis of familial form of these disorders [21]. Moreover, deregulation of the expression of diverse classes of ncRNAs, including circRNAs has been implicated in aging and in the pathophysiology of neuropsychiatric and neurodegenerative disorders [22,23,24]. 

In this review describes the current knowledge of circRNA biogenesis and function, the evidence supporting their specific activity in the nervous system and their putative implication in neurological disorders. Moreover, different aspects for circRNA-based therapy are discussed. 

## 2. Biogenesis of Circular RNAs 

**Figure 1:**
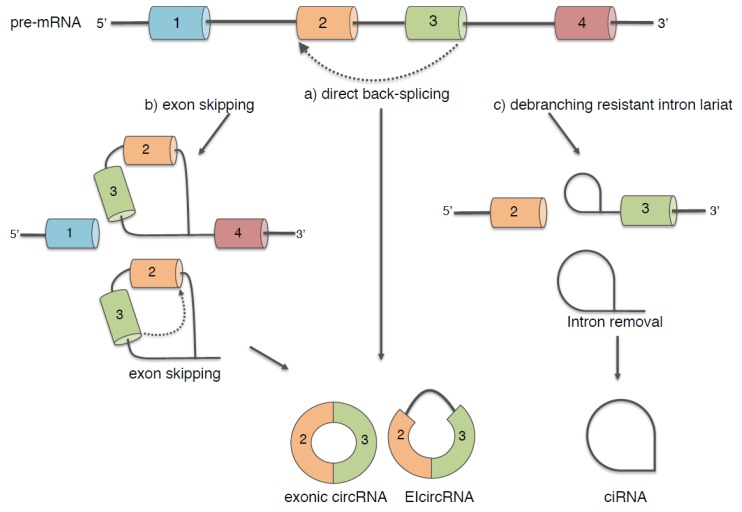
Mechanisms for circRNA biogenesis. CircRNA formation may proceed through a direct back-splicing pathway (dashed arrow) driven by intron pairing or RNA-binding protein interaction (**a**, see Figure 2), through an exon skipping lariat mechanism (dashed arrow) during alternative splicing events (**b**) and alternatively, circRNAs may be produced through the canonical splicing pathway depending on the presence of lariats resistant to debranching activity (**c**).

The vast majority of circRNAs are produced from protein-coding genes by different mechanisms: (i) direct back-splicing [25], a peculiar splicing reaction; (ii) exon skipping driven by a lariat structure [26] and (iii) debranching resistant intron lariat (Figure 1) [13]. 

These atypical splicing events still require canonical spliceosome machinery, they occur almost during transcription and in some cases in competition with the splicing of the linear host transcript [2,27].

However, at least for the direct back-splicing and exon skipping, the peculiarity resides in the ligation of a downstream 5′ donor splice site with an upstream 3′ acceptor splice site generating covalently closed circular transcripts. As a result of back-splicing and exon skipping events, mature circRNAs can consist of a single or multi- fully spliced exons (exonic circRNAs), exons with retained introns (exon intron circRNAs, EIcircRNAs) and UTRs.

A specific class of circRNAs, the so-called circular intronic circRNAs (ciRNAs), are instead produced by intron lariats that escape debranching. Therefore, in this case, ciRNAs accumulate in cells as a consequence of the canonical splicing reaction. It has been described that the resistance to the debranching activity relies on consensus RNA motifs near the 5′ splice site and the branchpoint [13]; however, the mechanism underlying this process is still unknown. 

CircRNA molecules produced by the all above-mentioned mechanisms lack 5′ and 3′ termini, thus, resulting highly resistant to degradation by exonucleases: the average half-life of circRNAs in cells is estimated to be ≈48 h [28], which is much longer than the average half-life of mRNAs (≈10 h) [29]. 

## 3. Regulation of Biogenesis of Circular RNAs

Although both direct back-splicing and exon skipping lariat mechanisms can take place *in vivo*, some evidence indicates that direct back-splicing may occur more frequently than exon skipping in higher eukaryotes [30].

**Figure 2:**
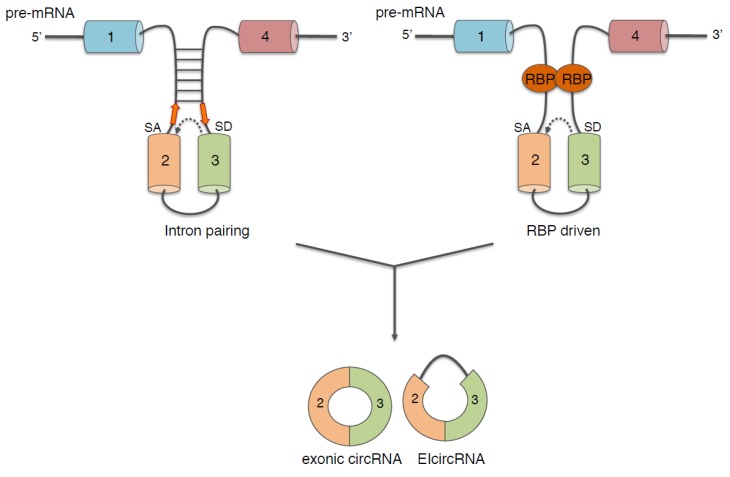
Features promoting circularization. Reverse complementary sequences (left; orange arrows indicate the reverse complementarity) and/or RNA-binding proteins (RBPs; right), act to bring the downstream splice-donor site (SD) into close proximity with an upstream splice-acceptor site (SA), thus, facilitating back- splicing reaction (dashed arrow).

The major requirement for the back-splicing reaction to occur is the proximity of the two splicing junctions; two elements have been described to play an important role in this mechanism: intronic *cis*-elements and protein factors acting in *trans* (Figure 2). 

Generally, it has been reported that long introns tend to be more prone in allowing the back-splicing reaction; indeed, highly expressed circRNAs have longer introns and genes giving rise circRNAs contains longer intronic sequences than genes that do not undergo back-splicing. Moreover, 20% of all genes producing circRNAs are expressed in brain, and it has been observed that genes with neuronal function and genes involved in axon guidance contain, on average, long intronic sequences [23,31].

Analyses of intronic sequences revealed that the presence of complementary inverted repeats in these long flanking introns positively correlates with the biogenesis of many circRNAs. Indeed, through base pairing, they allow the 3′ and 5′ splice sites of a single or two exons to be in spatial proximity [25,32,33]. On the contrary base pairing within one of the two flanking introns inhibits circularization suggesting the existence of competition for stem formation; therefore, dynamic changing in base pairing (between two flanking introns or inside one intron) might regulate the expression of specific circRNAs [33]. In this regard, it has been shown that the A-to-I RNA-editing enzyme ADAR, which has important functions during the development and function of the nervous system [34,35], can bind and edit the complementary inverted sequences. This event occurs more frequently at positions that were proximal to the splice sites involved in the circularization, thus, impairing the formation of intronic base pairing and inhibiting circRNAs production [36]. Notably, intronic repeats flanking circRNAs are enriched in editing sites suggesting an important regulatory role of ADAR in controlling circRNAs biogenesis.

In humans, ALU sequences are the repetitive elements contributing the most to circRNAs biogenesis [28]; indeed, 88% of human circularising exons has shown to contain inverted ALU repeats in flanking introns [36]. A recent study showed that the circularization of such exons could be affected by the activity of the DHX9 helicase, that is able to bind and melt double stranded RNAs formed by inverted ALU repeats [37]. Notably, DHX9 also interacts with ADAR and ALU elements are the main targets of this latter enzyme [37,38], altogether suggesting a synergistic effect of these two enzymes on the processing of ALU containing transcripts [37]. Moreover, Osenberg and co-workers showed that when human embryonic stem cells (hESCs) were differentiated toward neuronal fate, editing of inverted ALU repeats by ADAR1 decreased [39] and this well correlates with the overall upregulation of circRNAs observed during neuronal differentiation in both flies and mammals [3].

Is interesting to note that deregulation or mutations of DHX9 and ADAR enzymes have been associated with aging, immune disorders, several types of cancers and neurological diseases [40,41], implying deregulation also of circRNAs expression in all these pathological conditions.

Differently from ADAR1 and DHX9, that antagonize circRNAs biogenesis by counteracting intronic base pairing, the immune response factor NF110/90 binds and stabilizes transient RNA pairs formed between intronic complementary sequences, thus, promoting circRNAs biogenesis [42]. Li and co-workers observed that circRNAs production is decreased upon virus infection and correlated this phenomenon with the cytoplasmic NF110/90 translocation occurring in this condition [42]. 

The intronic inverted repeats are structural elements that are present in the flanking introns regardless of the cell type; therefore, their presence is not sufficient to explain the diverse and dynamic pattern of expression of circRNAs. It has been reported that the back-slicing reaction can be facilitated, and also modulated, by the activity of specific RBPs, which, instead, shows a spatial- and temporal-specific expression pattern. This better explains the specific expression of circRNAs in different cell types and under different pathophysiological circumstances. The molecular mechanism proposed for facilitating circularization relies on the binding of RBPs to flanking introns and on their dimerization, thereby allowing the two splice junctions to be in close proximity [2,43,44]. Experimental evidence demonstrated that muscle blind (MBL) in Drosophila and Quaking (QKI) and Fused in sarcoma (FUS) in mammals are directly involved in promoting back-splicing reaction [2,43,44]. These proteins act in a sequence specific manner; therefore, they can regulate the biogenesis only of circRNAs possessing these motifs in their flanking introns. The activity of RBPs is essential for neuronal development, and often, the impairment of their functions is associated with neurological disorders. For instance, deficiency of QKI may contribute to schizophrenia and to the development of inherited ataxia [45,46], whereas mutations of FUS gene are linked to the pathogenesis of familial form of ALS [47,48]. By this matter, it has recently been shown that the biogenesis of specific circRNAs is impaired both in murine and human *in vitro* derived motor neurons lacking the FUS gene or carrying FUS mutations [44] (Morlando personal communication).

## 4. Functions of Circular RNAs

Although only the functions of few circRNAs have been uncovered so far, a growing number of studies has revealed that circRNAs are involved in a wide range of cellular processes, as well as in human pathologies strongly suggesting their potential role as major regulators of gene expression.

### 4.1. Circular RNAs as microRNA and RBP “sponges”/scaffold

**Figure 3:**
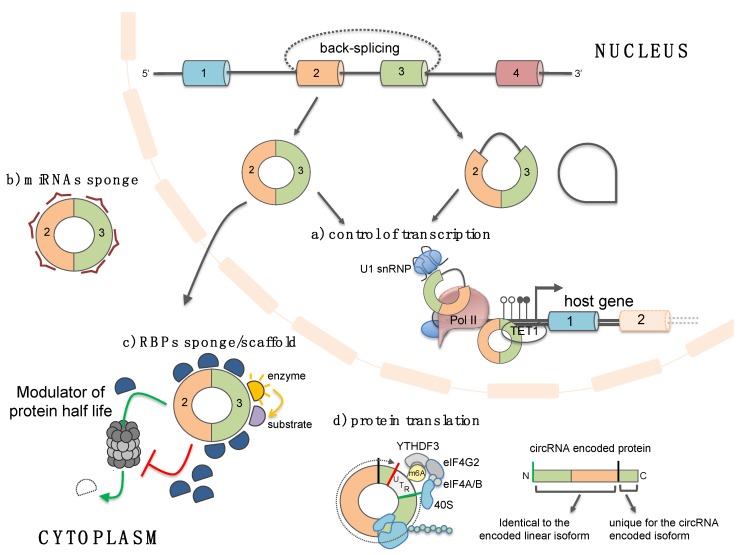
CircRNA functions. CircRNAs localized in the nucleus can function as a modulator of transcription of their host genes either by interacting with U1 small nuclear ribonucleoprotein (U1 snRNP) and enhancing the function of RNA polymerase II (Pol II) complex or by recruiting methylcytosine dioxygenase TET1 to the promoter region (**a**). When exported into the cytoplasm circRNAs can function as sponges or decoys for microRNAs and RBPs or alternatively can modulate the half-life of specific RBPs counteracting (red T line) or favoring their proteasome mediated degradation (green arrow) (**b**,**c**). CircRNAs have been shown to function also as protein scaffolds (**c**). By facilitating the colocalization of enzymes and their substrates are able to enhance the reaction kinetics (yellow arrow). Finally, circRNAs with internal ribosome entry site (IRES) elements and AUG sites (green line) may be translated through a CAP-independent mechanism (dashed arrow; red line depicts the STOP codon). This latter is promoted by the presence of methyl adenosine (m6A) and by the involvement of the reader protein YTHDF3 and the IRES-specialized translation initiation factor eIF4G2 (**d**). The protein isoform produced from circRNA translation will have part of the primary sequence in common with the linear encoded protein, while the rest of the polypeptide is unique for the circRNA encoded isoform.

Several studies have characterized a number of circRNAs that possess miRNA recognition elements (MREs) and through interaction with miRNA-Ago2 complexes act as effective “sponges”, thus, altering the expression of the natural miRNA targets (Figure 3a) [5,6,7,8]. A prime example is the cerebellar degeneration-related antigen 1-antisense circRNA, CDR1-AS. This circRNA, highly expressed in the mammalian brain and upregulated during neuronal development [3], has more than 70 sites for miR-7, most of them conserved across eutherian mammals [5,6]. The high number of MREs, together with the fact that CDR1-AS is much more expressed of any other housekeeping gene in mouse and human brain, suggests that the competing activity for miR-7 binding is stoichiometrically relevant in neuronal tissue [5]. Indeed, in zebrafish, which expresses mir-7, but not CDR1-AS, the ectopically expression of this circRNAs causes defects in midbrain development, phenocopying the miR-7 knock-down [6]. Intriguingly, mir-7 has been implicated, as a key regulator, in different cancers [49] and in neurological disorders, such as PD. The PD related α-synuclein is, indeed, a target of miR-7. The implication of CDR1-AS-miR-7 axis in the nervous system physiology and pathology is discussed below. 

Besides CDR1as, only few circRNAs are highly expressed and can function efficiently as miRNAs sponges; two examples are the circular Sry, which has 16 binding sites for miR-138 in mouse (but only one in human) [5], and circ-HIPK3, which, instead, has 18 putative binding sites for nine different miRNAs [7]. Therefore, it is not surprising that this field is very debated: the majority of the circRNAs described as “sponges” were indeed found to have only a single or very few binding sites for miRNAs raising the doubt regarding the effectiveness of their sponge activity [50,51]. 

In addition to miRNAs, circRNAs can bind to RBPs and sequester them from their natural targets or regulate their activity/stability (Figure 3b) [2,9,10,11]. Indeed, some circRNAs act as protein scaffolds favoring the colocalization of specific enzymes with their substrates [9,52]. CircMBL harbors numerous binding sites for the MBL protein that can, in turn, promotes the biogenesis of circMBL at the expenses of the production of the mature linear MBL mRNA. Therefore, it has been suggested that circMBL, by sequestering MBL, acts in a regulatory loop to finally fine tune the production and availability of the MBL protein [2]. Circ-FOXO3 and circ-ZNF609, are instead involved in controlling cell proliferation by inhibiting or promoting, respectively, the proteasome mediated degradation of specific cell cycle-related proteins [9,11]. In particular, circ-FOXO3 has been found to inhibit tumor genesis and progression and to be down-regulated in breast cancer, while an upregulation of circ-ZNF609 was described in Rhabdomyosarcoma [11,53,54]. The mechanism of action of circ-FOXO3 has been clarified: it has been described to act as a scaffold for mouse double-minute 2 (MDM2) and p53, thus, favoring the MDM2-dependent ubiquitylation of p53 [9]. Finally, a specific subgroup of circRNAs sharing 16–26 bp intra-double stranded RNA regions has been recently identified and shown to bind to PKR (dsRNA-activated protein kinase), thus, counteracting its activation in normal cultured cells. Liu and co-workers demonstrated that, upon viral infection, the activation of the endonuclease RNase L is responsible for circRNAs degradation and that this event is required for PKR release and activation in early cellular innate immune response [10]. Moreover, even though at its early stages, the work also revealed a correlation between a reduction of circRNAs expression and a stable activation of RNase L and PKR in patients with autoimmune disease systemic lupus erythematosus (SLE).

### 4.2. Circular RNAs as Templates for Protein Translation

Although generally considered “noncoding” molecules so far, circRNAs may hold the ability to serve as templates for protein translation (Figure 3c). This implies that new reading frames generated through circRNAs translation would expand the repertoire of protein isoforms in cells. Abe and colleagues were among the first to report the possibility for a circRNA molecule to be translated, revealing a rolling circle translation in rabbit reticulocyte lysate of an artificial circRNA with infinite open reading frame (ORF) [55]. Several other following studies demonstrated that endogenously produced circRNAs are indeed associated with polysomes and shifted to lighter fractions upon puromycin treatment [15,16,17]. Due to their circularity, circRNAs translation relies on a CAP-independent mechanism; moreover, it has been demonstrated that, *in vivo*, circRNAs must experience splicing to be competent for translation [16]. Further supports to a CAP-independent translation come from the work of Yang and colleagues which revealed that human circRNAs contain extensive m^6^A modifications; this latter was shown to promote CAP-independent circRNAs translation through the involvement of the reader protein YTHDF3 and the IRES-specialized translation initiation factor eIF4G2 [17]. To date, only for a handful number of circRNAs the function of the translated protein isoform has been determined [18,19,20]. Nevertheless, the fact that the CAP-independent translation is enhanced in stress condition provides an interesting clue for the possibility that circRNAs-encoded proteins may play roles in a particular cellular condition, such as stress response.

### 4.3. Circular RNAs Regulate Gene Transcription

The regulation of gene expression through the miRNAs and RBPs sponge activity of circRNAs has been widely studied, since the majority of the identified circRNAs are localised in the cytoplasm; however, circRNAs have been reported to be also localised in the nucleus where they control gene expression at the transcriptional level (Figure 3d). For instance, two nuclear-localised circRNAs, that retain an intron (exon-intron circRNAs, EIcircRNA), circEIF3J and circPAIP2, through interactions with U1 small nuclear RNA (snRNA), the RNA polymerase II (RNAPII) and promoter regions are able to facilitate the expression of their parental genes [12]. The same function has been described for ci-ankrd52 and ci-sirt7, two intronic circRNAs; it has been demonstrated that they accumulate at the site of active transcription and through interaction with elongating RNAPII modulate the rate of transcription of their parental genes [13]. Fully spliced exonic circRNAs have also been detected in the nucleus [14,44]. One example is FECR1 circRNA that regulates the *FLI1* gene by binding to the promoter region and by recruiting TET1 DNA demethylase to induce DNA demethylation [14].

## 5. Circular RNAs Expression and Function in the Nervous System and Neurological Diseases

To date, several studies have reported that circRNAs are not ubiquitously expressed, whereas they result specifically enriched in the nervous system and accumulate with age [2,3,4,23,24]. This can be explained by the importance of the alternative splicing in this system, which contributes the most to the morpho-functional complexity of neuronal cells [56], and by the fact that the majority of circRNAs producing genes are expressed in brain. Additionally, the high stability of circRNAs might have a great impact on their accumulation in post mitotic tissues. However, in some cases when the host gene is also expressed in other tissues, the transcripts undergoing back-splicing in the brain is significantly higher [4], suggesting the presence of a neuronal-specific regulation of circRNAs biogenesis. Moreover, circRNAs show regulated expression in various brain regions and subcellular compartments both during embryogenesis and at postnatal stage [4,6,31,57].

Notably, among the neuronal genes hosting circRNAs, a conspicuous group encodes for proteins involved in synaptogenesis and synaptic function [2,3,4,23]. Moreover, circRNAs arising from these genes (e.g., Dscam, Homer and Stau2) are enriched in dendrites, differently from their linear counterparts, suggesting an active transport of these RNAs from the cell body to synapses [3,4]. Additionally, You and colleagues demonstrated that the expression and the accumulation in dendrites of dozen of circRNAs (e.g., circ-Homer was the most significantly upregulated), but not of the linear counterparts, are modulated by the induction of homeostatic synaptic plasticity in cultured neurons [4]. Altogether these findings strongly suggest a specific function of circRNAs in synapses and allow intriguing hypothesis on the functional significance of the synaptic localization of circRNAs: they could serve as scaffolds to assemble RNAs and proteins and to deliver them to synapses or alternatively they could be packaged into vesicles and released at synaptic levels serving as messaging molecules between cells. Some neuronal mRNAs are localised and translated in dendrites and synapses, since, as described above, circRNAs can also be translated, it can be inferred that the regulated transport and translation of these circRNAs could contribute to the specific dendrites protein content affecting neuronal activity and plasticity. 

Modulation of circRNAs expression has also been linked to neurological diseases. In general, neurotoxic insults or neuronal damage induce changes in coding gene expression, a shift in the splicing profile and alteration of expression of ncRNAs, including circRNAs. Deregulation of CDR1-AS, the most studied and the most highly expressed mammalian circRNAs, has been linked to neuropsychiatric and neurodegenerative diseases, as well as to brain tumours [58,59,60]. As mentioned above, the CDR1-AS harbours more the 70 sites for miR-7 acting as an effective sponge for this miRNA. Notably, miR-7 is highly expressed in cortical neuron progenitors and its downregulation, by the use of artificial sponges, leads to microcephaly-like brain defects [61]. Additionally, alteration of the CDR1-AS-mir-7 axis, due to the downregulation of CDR1-AS, has been described in the hippocampus of AD patients [58]. These latter also exhibit downregulation of the ubiquitin protein ligase A (UBE2A), the protein responsible for the clearance of AD-amyloid peptides contributing to their accumulation and aggregation in the cytoplasm. Since UBE2A is a mir-7 target gene, its expression is most likely lowered in AD patient because of the reduced levels of CDR1-AS with the concomitant depletion of its sponge activity on miR-7 [62]. The involvement of CDR1-AS in AD is further support by *in vitro* experiments showing that the overexpression of this circRNA promotes degradation of APP and beta-secretase 1 (BACE1) through proteasomal and lysosomal pathways, thus, reducing the accumulation of AD-amyloid peptides [63].

Intriguingly, also α-synuclein, whose expression is implicated in the pathophysiology of PD, is a target of miR-7 [64,65]. Indeed, human cell lines transfected with miR-7 showed a reduced level of α-synuclein, and this outcome is lessened by the concomitant overexpression of CDR1-AS [5]. Moreover, miR-7 has also a neuronal protection function, since its expression counteracts cell death induced by the dopaminergic neurotoxic compound MPP (1-methyl-4-Phenyl-Pyridinium) through upregulation of mTOR pathway [66,67]. Therefore, it can be suggested that the CDR1-AS - mir-7 regulatory network might have a role also in PD. 

miR-671 is another miRNA that binds to CRD1-AS; however, differently from mir-7, it binds only once and with a perfect complementarity, thus, inducing CRD1-AS Ago2-mediated degradation [68]. This, together with the evidence of CDR1-AS localization in neuronal processes, opens the possibility that, beside its sponge activity, CDR1-AS might work as a cargo for miR-7 and that regulation of miR-671 expression in time and space (e.g., upon stress or in dendrites) would allow its release; for instance, miR-671 might be modulated in response to different stress conditions or in specific cell sub-compartments, such as neuronal processes, in order to locally control the expression of mRNAs, targets of miR-7. All of this might be impaired in neurological diseases. 

Lastly, Piwecka and co-workers, in a recent study, showed that CRD1-AS knockout mice display prepulse inhibition of the startle response, a sensorimotor gating phenotype that is impaired in several human neuropsychiatric disorders. Moreover, neurons from these mice have increased spontaneous vesicle release suggesting that CRD1-AS might play a role in regulating synaptic transmission [59].

In the last few years, a complex link between circRNAs and neurodegenerative disorders has emerged: transcriptome analyses through microarray and RNA-sequencing revealed that deregulation of many other circRNAs is associated with AD [69,70], multiple system atrophy [71] and ALS [44]. In particular, Errichelli and co-workers showed that in cultured murine motor neurons lacking the FUS gene, whose mutation is linked with 5% of familial ALS, hundreds of circRNAs resulted deregulated, despite the unaltered accumulation of their linear counterparts. They also determined that FUS is able to impact directly on the biogenesis of specific circRNAs, either in a positive or negative manner, through the binding of intronic regions flanking exons involved in circularization. Intriguingly, circRNA deregulation is also observed in cultured human motor neurons carrying the P525L FUS mutation, which is linked to a severe and juvenile familial form of ALS [Morlando personal communication].

## 6. Circular RNAs as Neurological Disease Biomarkers and Therapeutics

Covalently closed ends endow circRNAs with high stability in blood, and other body fluids, this, together with their distinct and specific expression, makes circRNAs potential biomarkers for neurological diseases. Memczak and colleagues [72] were among the first to describe the presence of circRNAs in the blood and they have been followed by other groups detecting circRNAs in other body fluids, as well as in exosomes and extracellular vesicles [73,74,75,76,77,78]. In particular, Lasda and Parker found that circHIPK3, circZKSCAN1, circASXL1 and circKIAA0182 are secreted in extracellular vesicles, including exosomes and microvesicles, from different human cell lines, and are enriched in comparison with their respective linear RNAs. Lasda and Parker proposed that this might be a clearance mechanism to control the cellular level of such stable RNA molecules or that circRNAs are packaged into EVs for the purpose of cell to cell communication [78]. 

Since extracellular vesicles/exosomes are also potential biomarkers for neurological diseases, such as AD [79], the correlation between the expression of exosomal proteins, miRNAs, and circRNAs could increase the sensitivity and specificity of diagnosing multiple diseases, as well as assessing responses to drug treatments. 

CircRNAs have been already proposed as biomarkers for several diseases, including lupus erythematosus [80], tuberculosis [81,82], rheumatoid arthritis [83], diabetes [84,85], and cancer [77,80,86]. Concerning the use of circRNAs as biomarkers in neurological diseases the only example, so far, is the has_circRNA_103636 found to be differentially regulated in patients with major depressive disorder following an eight weeks antidepressant treatment [87]. This suggests that circRNAs can be used to assess responses to drug treatments, as well as diagnostic molecules. Due to the inaccessibility of the brain or spinal cord tissues for biopsy, for neurological diseases is really important to identify biomarkers in tissues obtained through non-invasive techniques. Is interesting to note that, the blood-brain barrier might be compromised in diseases, such as AD, PD and ALS [88] allowing free or micro vesicle encapsulated circRNAs to move towards the peripheral circulation. Therefore, it might be possible to correlate the blood levels of neuronal-specific circRNAs with the type of diseases, with its progression and eventually with the response to treatments.

Since deregulation of specific circRNAs has been linked to a defined disease, another interesting possibility is to consider such circRNAs as targets for the disease treatment. Indeed, it has been shown that manipulation of the abundance of specific circRNAs though siRNA mediated knockdown or ectopic expression has therapeutic value in *in vivo* models of cardiovascular disease [89]. Moreover, Armakola and colleagues [90] found that ciRNAs, accumulating in the cytoplasm upon knockdown of debranching enzyme 1 (Dbr1) activity, sequestered TDP-43 in this cellular compartment and suppressed its toxicity in the human neuronal cell line and primary rat neurons. Since the cytoplasmic accumulation of the RBP TDP-43 has been found in some forms of sporadic ALS [91] the modulation of ciRNAs biogenesis through inhibition of Dbr1 function was suggested as a potential therapeutic strategy for ALS [90].

Besides the modulation of native circRNAs, circular transcripts themselves might be used as therapeutic agents. For instance, the circular backbone can be engineered in order to generate artificial high stable transcripts with customised molecular effects: sponges for specific miRNAs or RBPs, templates for translation of specific peptides, modulators of immune response or transcription/splicing regulators.

Regardless of the use of circRNAs as therapeutic agents or targets, one limitation might be the methods for delivering therapeutic nucleic acids to CNS. Nevertheless, emerging strategies for molecules design and modification, types of administration and delivery to the CNS have been described and actively used *in vivo*. Antisense oligonucleotides (ASOs) have been administrated to the CNS via intracerebroventricular or intrathecal infusion of CSF into rodent and non-human primate animal models for tauopathy, HD, ALS and spinal muscular atrophy (SMA) [92,93,94,95,96]. More importantly, clinical trials in patients with ALS or SMA that involve intrathecal delivery of ASOs against protein-coding transcripts have demonstrated the promise of therapeutically targeting RNAs [97,98,99].

Starting for these existing effective approaches, a circRNAs-based therapy can be foreseen for the treatment of neurodegenerative disorders.

## 7. Perspectives

The fast advance in next-generation RNA sequencing and bioinformatics allowed the discovery of thousands of circRNAs which are challenging our understanding of gene expression regulation. While circRNAs are becoming a promising field of study, we can state that we are in the early days, since there are still many open questions on these peculiar non-coding RNA molecules. In particular, little is known about the regulation of their biogenesis in different tissues and under physiological/ pathological conditions, and even less about their function: the vast majority of annotated circRNAs still waits for functional characterization. However, many features of these molecules, such as their high stability, neuronal-specific expression and their modulation during neuronal development and activity, hold great promise for having an important impact in the context of neurodegeneration. Further investigation will be needed in order to identify specific circRNAs associated with defined neurological diseases and to determine how circRNAs control cellular pathways in neurons. This will help us to better understand the heterogeneity and the dynamic aspects of neurological disorders and might offer the opportunity to explore effective new approaches for therapeutics. 

## Figures and Tables

**Table 1:** List of housekeeping and regulatory non-coding RNAs (ncRNAs) together with the corresponding abbreviation and function.

Full Name	Abbreviation	Function
*Housekeping ncRNAs*		
Ribosomal RNA	rRNA	Translation machinery
Transfer RNA	tRNA	amino acid cargo; decoding the genetic codes
Small nuclear RNA	snRNA	RNA processing
Small nucleoar RNA	snoRNA	RNA modification
Telomer RNA	TERC	Telomere maintenance
*Regulatory ncRNAs*		
microRNA	miRNA	post-transcriptional control
DNA damage response RNAs	DDRNAs	DNA damage repair
Repeat-derived RNA	rasiRNA	Transcriptional control
Endogenous siRNA	endo-siRNA	Transposon silencing and pseudogene expression
Piwi-associated RNA	piRNA	Transposon silencing and mRNA decay
Enhancer RNA	eRNA	Transcriptional control
Promoter associated RNA	PAT	Transcription initiation and pause release
Long non-coding RNA	lncRNA	Transcriptional and post-transcriptional control
Circular RNA	circRNA	Transcriptional and post-transcriptional control
